# A comparative study of the efficacy of apitoxin gel versus photobiomodulation by diode laser for recurrent aphthous stomatitis treatment

**DOI:** 10.1016/j.jtumed.2026.09.007

**Published:** 2026-10-01

**Authors:** Mohamed Omar Elboraey, Sherouk Mohamed Mohamed Gamal, Reda Saber Sabra, Mostafa I. Fayad, Abdullah Alqhtani, Rayan Meer

**Affiliations:** a Periodontology, Oral Diagnosis and Oral Radiology Department, Faculty of Dentistry, Tanta, University, Tanta, Egypt; b Department of Preventive Dental Sciences, College of Dentistry, Taibah University, Al-Madinah Al-Munawwarah, KSA; c Department of Substitutive Dental Science, College of Dentistry, Taibah University, Almadinah Almunawwarah, KSA

**Keywords:** طب الأسنان, العلاج بسم النحل, الأبيتوكسين, التهاب الفم القلاعي الناكس, العلاج بالتعديل الحيوي الضوئي, Apitoxin, Bee venom therapy, Dentistry, Photobiomodulation therapy, Recurrent aphthous stomatitis

## Abstract

**Background:**

Bee venom (apitoxin) contains bioactive compounds. Diode laser irradiation has a photobiomodulation effect. Both modalities are considered potential alternative therapies for managing recurrent aphthous stomatitis (RAS).

**Objective:**

This study compared the therapeutic effectiveness and safety of apitoxin gel versus photobiomodulation therapy (PBM) for the management of recurrent aphthous ulcers.

**Methods:**

Sixty participants with symptomatic recurrent aphthous ulcer ≥4 mm were randomly allocated to two groups. Group I was managed by PBM and group II by using 0.3% apitoxin gel. The pain sensation score measured using a visual analog scale (VAS), and the ulcer surface area and ulcer depth were recorded at baseline and on day 5. The effectiveness index (EI) for pain improvement was calculated for each patient.

**Results:**

The Shapiro–Wilk test was applied to verify the normality of distributions using IBM SPSS software package version 24.0. The independent-samples *t*-test was used to compare differences between two means. Significant improvements in all clinical parameters were found post-treatment in both study groups compared with the baseline (paired *t*-test, *P* < 0.001). According to the intergroup comparison, the clinical improvement was greater in group II, and significant differences in pain sensation (VAS), the EI for pain reduction, and ulcer depth were found between the two groups (independent-samples *t*-test, *P* < 0.001). The ulcer surface area did not differ significantly between the two groups post-treatment (independent-samples *t*-test, *P* = 0.21).

**Conclusion:**

Apitoxin gel reduces pain, speeds healing, and controls inflammation in RAS. Its effects are due to anti-inflammatory, antioxidant, and antimicrobial activities, and it may provide a more cost-effective and convenient option for patients.

**Trial registration:**

ClinicalTrials.gov, TRN: NCT07135609.

## الملخص

**أهداف البحث:**

يحتوي سم النحل (الأبيتوكسين)على مركبات حيوية فعّالة، كما يمتلك التشعيع بليزر الصمام الثنائي تأثيرًا في التعديل الحيوي الضوئي. وتُعد كلتا الطريقتين من الخيارات العلاجية البديلة المحتملة لتدبير التهاب الفم القلاعي الناكس. هدفت هذه الدراسة إلى مقارنة الفعالية العلاجية والسلامة بين هلام سم النحل والعلاج بالتعديل الحيوي الضوئي في تدبير القرح القلاعية الناكسة.

**طرق البحث:**

شملت الدراسة 60 مشاركًا يعانون من قرحة قلاعية ناكسة مصحوبة بأعراض، بقطر لا يقل عن 4 مم، وجرى توزيعهم عشوائيًا إلى مجموعتين. عولجت المجموعة الأولى بالتعديل الحيوي الضوئي، بينما عولجت المجموعة الثانية باستخدام هلام سم النحل بتركيز 0.3%. قُيّمت شدة الألم باستخدام المقياس التناظري البصري للألم، كما سُجلت مساحة سطح القرحة وعمقها عند خط الأساس وفي اليوم الخامس. وحُسب مؤشر الفعالية لتحسن الألم لكل مريض.

**النتائج:**

استُخدم اختبار شابيرو–ويلك للتحقق من التوزيع الطبيعي للبيانات باستخدام الحزمة الإحصائية IBM SPSS، الإصدار 24.0. كما استُخدم اختبار ت لعينتين مستقلتين لمقارنة الفروق بين المتوسطات. أظهرت كلتا مجموعتي الدراسة تحسنًا ذا دلالة إحصائية في جميع المؤشرات السريرية بعد العلاج مقارنة بخط الأساس اختبار ت للعينات المترابطة، (P < 0.001). وعند المقارنة بين المجموعتين، كان التحسن السريري أكبر في المجموعة الثانية، مع وجود فروق ذات دلالة إحصائية بين المجموعتين في شدة الألم وفق المقياس التناظري البصري، ومؤشر فعالية خفض الألم، وعمق القرحة (اختبار ت لعينتين مستقلتين، P < 0.001) ). في المقابل، لم يظهر اختلاف ذو دلالة إحصائية بين المجموعتين في مساحة سطح القرحة بعد العلاج اختبار ت لعينتين مستقلتين، P = 0.21)).

**الاستنتاجات:**

يسهم هلام سم النحل في تخفيف الألم، وتسريع التئام القرح، والسيطرة على الالتهاب في حالات التهاب الفم القلاعي الناكس. ويُعزى تأثيره إلى خصائصه المضادة للالتهاب، والمضادة للأكسدة، والمضادة للميكروبات، وقد يمثل خيارًا علاجيًا أكثر ملاءمة وأقل تكلفة للمرضى.

## Introduction

Recurrent aphthous stomatitis (RAS) is a recurrent idiopathic, inflammatory round or oval-shaped oral ulceration with erythematous borders.^1^ Due to its painful nature, RAS can significantly affect quality of life by interfering with daily activities.^2^^,^^3^ The primary objectives in the management of RAS are pain elimination, healing improvement, and prevention of recurrence.

The conventional treatment options for RAS include various topical and systemic pharmacological agents such as corticosteroids, antiseptics, and anti-inflammatory medications. The treatment usually starts with topical applications, and systemic therapy is mainly reserved for cases with severe symptoms or frequent episodes. However, the effectiveness of these treatments is sometimes limited by side effects or insufficient impact on the recurrence rate.^4^^,^^5^

Topical corticosteroids remain the cornerstone of RAS management, providing localized anti-inflammatory effects with minimal systemic absorption.^3^ Several side effects such as oral candidiasis can occur due to the frequent or prolonged use of corticosteroids.^6^ Recently, photobiomodulation therapy (PBM) has gained attention as an alternative treatment for RAS. PBM is effective at modulating inflammation, promoting tissue regeneration, enhancing healing, and providing pain relief. These benefits are attributed to its ability to activate cellular proliferation, regulate inflammatory mediators, and improve tissue oxygenation.^7^

PBM is a non-thermal light therapy based on low-intensity laser or light-emitting diode irradiation within the red to near-infrared spectrum (600–1100 nm) to modulate cellular activities and promote tissue repair.^8^, ^9^, ^10^ The therapeutic effects of PBM are attributed to mitochondrial stimulation, elevated adenosine triphosphate (ATP) synthesis, and regulating reactive oxygen species, leading to reduced inflammation and accelerated healing.^11^ PBM has been successfully applied in the management of various oral mucosal conditions, including RAS, as a non-invasive and well-tolerated treatment modality.^12^^,^^13^

Recently, there has been a resurgence of interest in traditional medicine, particularly the use of bee products. Bee venom (BV), or apitoxin, is notable because of its broad range of biological effects, and it has been used for centuries in Oriental medicine.^14^^,^^15^

Apitoxin has attracted significant attention recently due to its multifaceted biological properties, which make it a promising choice for treating inflammatory conditions, including oral ulcers. The primary bioactive component, melittin, exhibits a potent downregulating anti-inflammatory effect on tumor necrosis factor-α and interleukin 6, while also concurrently inhibiting nuclear factor κB activation, a central mediator of inflammatory pathways**.**^15^^,^^16^ In addition, apitoxin contains apamin, a neuroactive peptide that modulates immune signaling through the nervous system, thereby further contributing to the suppression of inflammation and pain.^17^

The therapeutic potential of apitoxin is particularly advantageous in the management of oral ulcers, where excessive inflammation and pain are the primary clinical challenges. The antimicrobial properties of apitoxin, primarily mediated by melittin and phospholipase A2, play crucial roles in preventing secondary infections within ulcerated tissues, thereby enhancing wound healing and reducing complications.^14^ This synergistic integration among anti-inflammatory, analgesic, and antimicrobial mechanisms potentially positions apitoxin as a comprehensive and biologically driven therapeutic agent for oral ulcer management.

In addition to these properties, apitoxin actively facilitates tissue regeneration, which is a pivotal factor in ulcer healing. Research indicates that apitoxin enhances the proliferation of fibroblasts and keratinocytes, which are both integral to the restoration and repair of damaged oral mucosa.^18^ This effect is mediated by the activation of growth factors and enhancement of localized blood flow to accelerate tissue regeneration in the affected area.^19^

Unlike conventional therapies such as topical corticosteroids or antiseptics, apitoxin acts via multiple modes to simultaneously target inflammation, pain, infection, and tissue repair, making it a comprehensive therapeutic option. Due to its natural origin and cost effectiveness, apitoxin is a compelling alternative to synthetic drugs, offering comparable therapeutic benefits with minimal systemic side effects when administered at appropriate doses.^20^ These attributes highlight the potential of apitoxin as a safe and effective intervention for oral ulcers, necessitating further clinical research to refine and optimize its therapeutic application.

BV therapy involves the use of BV either through direct bee stings or injections of venom extracted through electrical stimulation. Historical observations that beekeepers who are frequently stung by bees rarely suffer from arthritis or other joint and muscle disorders have spurred interest in the therapeutic potential of BV.^21^^,^^22^ Several conditions have traditionally been treated with BV, such as arthritis, rheumatism, back pain, cancer, and skin diseases, as it activates different immune system functions via the increased production of cortisol.^23^, ^24^, ^25^, ^26^

RAS is the most common painful ulcerative disorder of the oral mucosa and it occurs worldwide. Even small ulcers (1–2 mm) can cause intense pain and recur repeatedly. This disorder poses a substantial challenge, and there is a clear need for research to identify therapies that both alleviate symptoms and reduce recurrence.^3^ The aim of this study was to evaluate the therapeutic efficacy of a novel apitoxin gel formulation compared with PBM in the management of RAS.

## Materials and Methods

### Study design

This randomized clinical study was ethically approved under approval number TUCDREC/300424/Rsabra. All procedures followed the ethical principles of the Helsinki Declaration. Prior to participation, informed consent was obtained from each patient (or from their parents or legal guardians if aged under 18 years) after receiving comprehensive information regarding the nature, objectives, and procedures of the study. Patient data were anonymized, assigned unique ID numbers, and stored securely on password-protected systems accessible only to the research team. The results are reported in aggregate to ensure confidentiality.

Before initiating the study, the two examiners underwent extensive and standardized training and calibration to ensure consistency in applying the treatment protocols and different assessment techniques. The process involved theoretical and practical training sessions focused on diagnostic criteria, applying the treatment protocols, and competency in the use of ImageJ software (National Institutes of Health, Bethesda, MD, USA). Inter-examiner reliability was determined by using Cohen's kappa coefficient, and a minimum of κ = 0.80 was considered essential for inclusion in the study. Intra-examiner variability was reevaluated for 20% of cases for each examiner after a 2-week interval, and agreement was measured using the intraclass correlation coefficient.

### Sample size calculation

The primary endpoint of this randomized controlled trial was improvement in the pain associated with recurrent aphthous ulcers measured by using a visual analog scale (VAS) from baseline to day 5. Pain reduction was further quantified using an effectiveness index (EI). Secondary endpoints include changes in the ulcer surface area and ulcer depth over the same period.

The sample size was determined based on detecting a clinically meaningful difference in healing outcomes between the PBM group and apitoxin gel treatment group. The calculation used the following parameters:

Expected effect size (Δ): 3.5 days difference in healing time.

Standard deviation (σ): 4.5 days.

Significance level (α): 0.05 (Z = 1.96).

Power (1 – β): 80% (Z = 0.84).

Comparison: two independent means.

The calculation was performed using the following formula:

(1) $$n={\left(\frac{Z_{\alpha /2}+Z_{\beta }}{\Delta }\right)}^{2}\times 2\sigma^{2}$$

After substituting the parameters, the result was as follows:

(2) $$n={\left(\frac{1.96+0.84}{3.5}\right)}^{2}\times 2\times \left(4.5\right)\times \left(4.5\right)=25.92$$

Thus, 26 participants per group were required to ensure adequate statistical power. In total, 60 participants were ultimately recruited to allow for attrition and ensure balanced allocation between the two treatment arms.^6^

The original sample size calculation was based on the “healing time,” which was not directly measured in this study. This may have affected the formal power estimation, but the observed effect sizes and confidence intervals indicate that meaningful information on the reported outcomes (VAS scores/ulcer area) was obtained in this study.

### Participants

In total, 60 patients were recruited from the Oral Medicine and Periodontology Outpatient Clinic, Faculty of Dentistry, Tanta University, Tanta, Egypt.

The inclusion criteria consisted of male and female patients aged between 15 and 40 years, with no history of systemic medical conditions. Eligible participants were also non-smokers who presented with pain from RAS ulcers on the oral mucosa for two days or less. The ulceration prevalence and types across study groups.

Patients were excluded if they had oral mucosal ulcers unrelated to RAS, systemic diseases associated with RAS (e.g., Behçet's disease), or any prior exposure to laser therapy. In addition, female patients who were pregnant, lactating, or postmenopausal were excluded from the study.

### Material preparation: apitoxin gel

A novel apitoxin gel was prepared at the Faculty of Pharmacy, Tanta University, Egypt, and registered under patent number EG/P/2024/1420 at the Egyptian Patent Office. The gel was prepared by dissolving 0.3 g of powdered BV (from *Apis mellifera*) obtained from an apiary in Egypt in 100 mL of 2% (v/v) propylene glycol (Merck, Darmstadt, Germany). Carbopol Ultrez 30 (1.5% w/w; Lubrizol Advanced Materials, Wickliffe, OH, USA) was gradually added under continuous stirring at 1252 x g to obtain a uniform gel, which was stored at 4 °C until use.

Cytotoxicity was evaluated using an immortalized human oral epithelial cell line from the National Research Centre in Egypt (Dokki, Giza, Egypt). Cells were cultured in keratinocyte serum-free medium (Gibco, Thermo Fisher Scientific, Waltham, MA, USA) supplemented with epidermal growth factor (Gibco), bovine pituitary extract (Gibco), and 1% penicillin/streptomycin (Gibco) at 37 °C under a humidified 5% CO_2_ atmosphere. For the (3-(4,5-Dimethylthiazol-2-yl)-2,5-diphenyltetrazolium bromide (MTT) assay, 10,000 cells/well were seeded in 96-well plates and allowed to adhere for 24 h, before treating with serial dilutions of apitoxin gel (6.25%, 12.5%, 25%, 50%, and 100%). Culture medium alone was used as a negative control and 10% dimethyl sulfoxide (Sigma–Aldrich, Burlington, MA, USA) as a positive control.

### Study groups and treatment strategy

Participants were randomly allocated to either group I (PBM) or group II (apitoxin gel application) using a computer-generated randomization sequence. Random numbers were generated using the = RAND() function in Microsoft Excel (Microsoft Corporation, Redmond, WA, USA) to produce uniformly distributed values. These values were then utilized to assign participants to one of the treatment groups in a blinded manner. In addition, the = RANDBETWEEN() function was applied to generate discrete random integers within a predefined range to ensure balanced group allocation. To eliminate bias and maintain equitable baseline characteristics, an independent statistician supervised the randomization process. Moreover, to ensure transparency and reproducibility, the randomization sequence remained concealed from investigators until the final intervention assignments were determined. Both groups received standardized instructions on gel application and follow-up protocols, thereby ensuring consistent treatment administration and uniform outcome assessment throughout the study.

To minimize bias, outcome assessments were performed by an independent examiner who remained blinded to group allocation throughout the study. Participants were also blinded to their assigned intervention. Due to the distinct nature of the two treatment modalities, operator blinding was unfeasible, so two independent operators were assigned who each administered only one specific modality and were kept completely separated regarding the treatment protocols.

#### **Group I:** PBM therapy

Participants assigned to group I were treated using a gallium–aluminum–arsenide (GaAlAs) diode laser (T8 laser device; Elexxion AG, Radolfzell, Germany). The laser operated at a wavelength of 806 nm and power output of 60 mW, with an exposure time of 80 s, delivering an energy density of 6.3 J/cm^2^ per session. The laser beam was applied directly to the ulcer surface using a glass rod. Each participant received four treatment sessions, which were administered once daily from day 1 to day 4. The parameters and clinical protocol followed established PBM guidelines reported in previous studies.^27^ Illustrations of the PBM procedure and clinical outcomes after four days are presented in Figure 1, Figure 2, Figure 3.^27^ Illustrations of the PBM procedure and clinical outcomes after four days are presented in Figure 1, Figure 2, Figure 3.^27^

**Figure 1:**
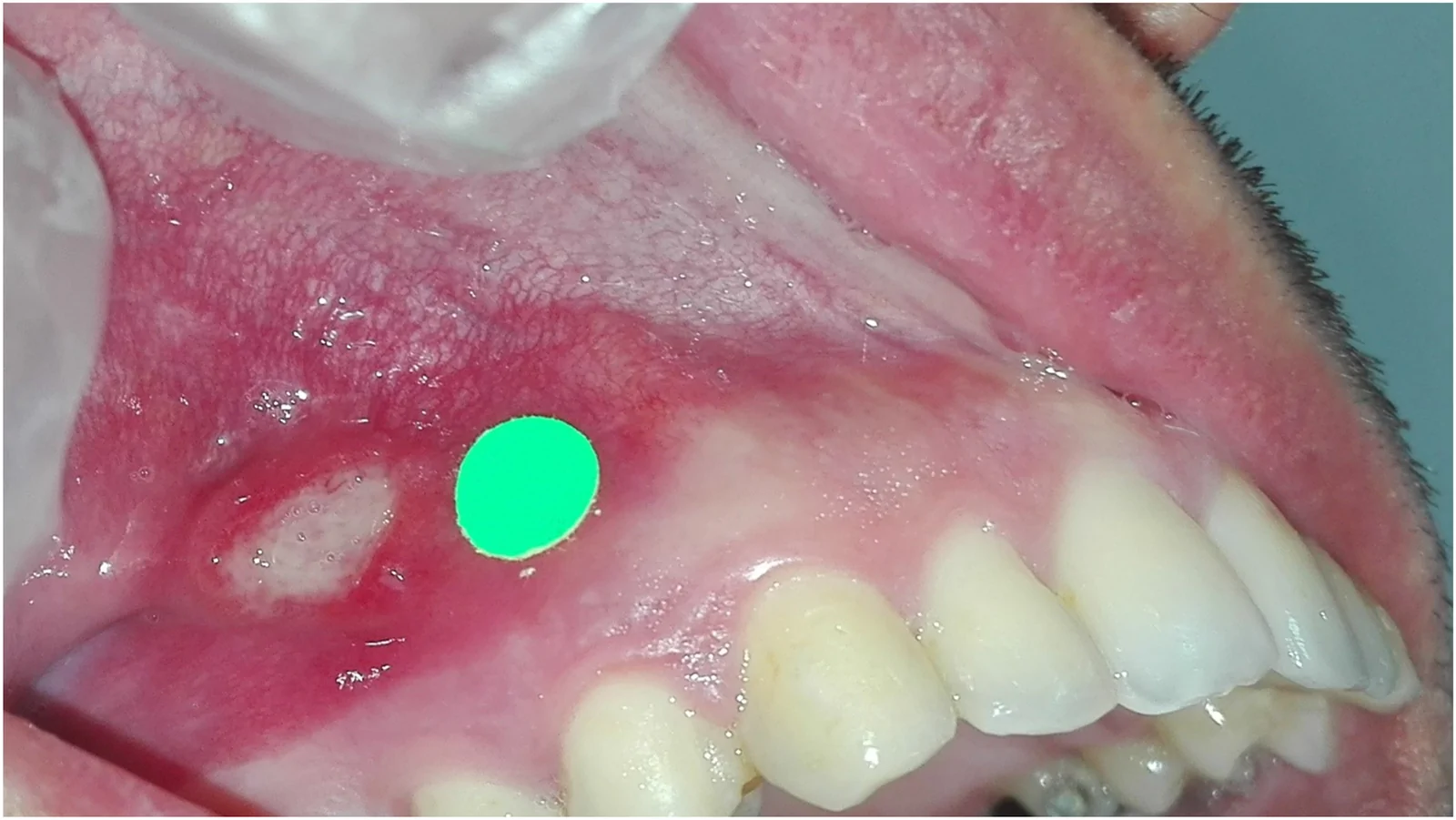
Recurrent aphthous stomatitis before low-level laser (photobiomodulation) therapy application.

**Figure 2:**
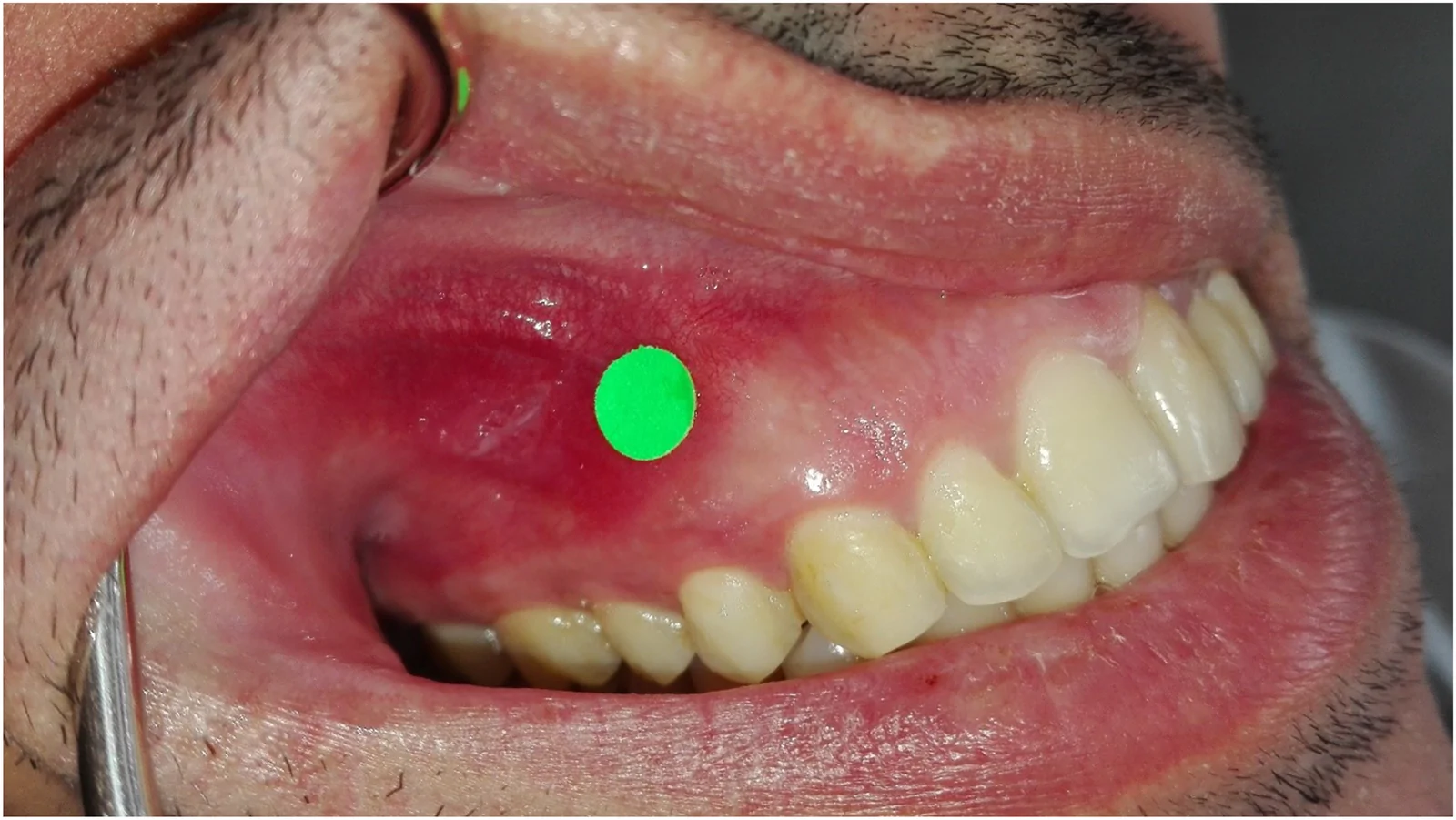
Effect following photobiomodulation therapy for recurrent aphthous stomatitis.

**Figure 3:**
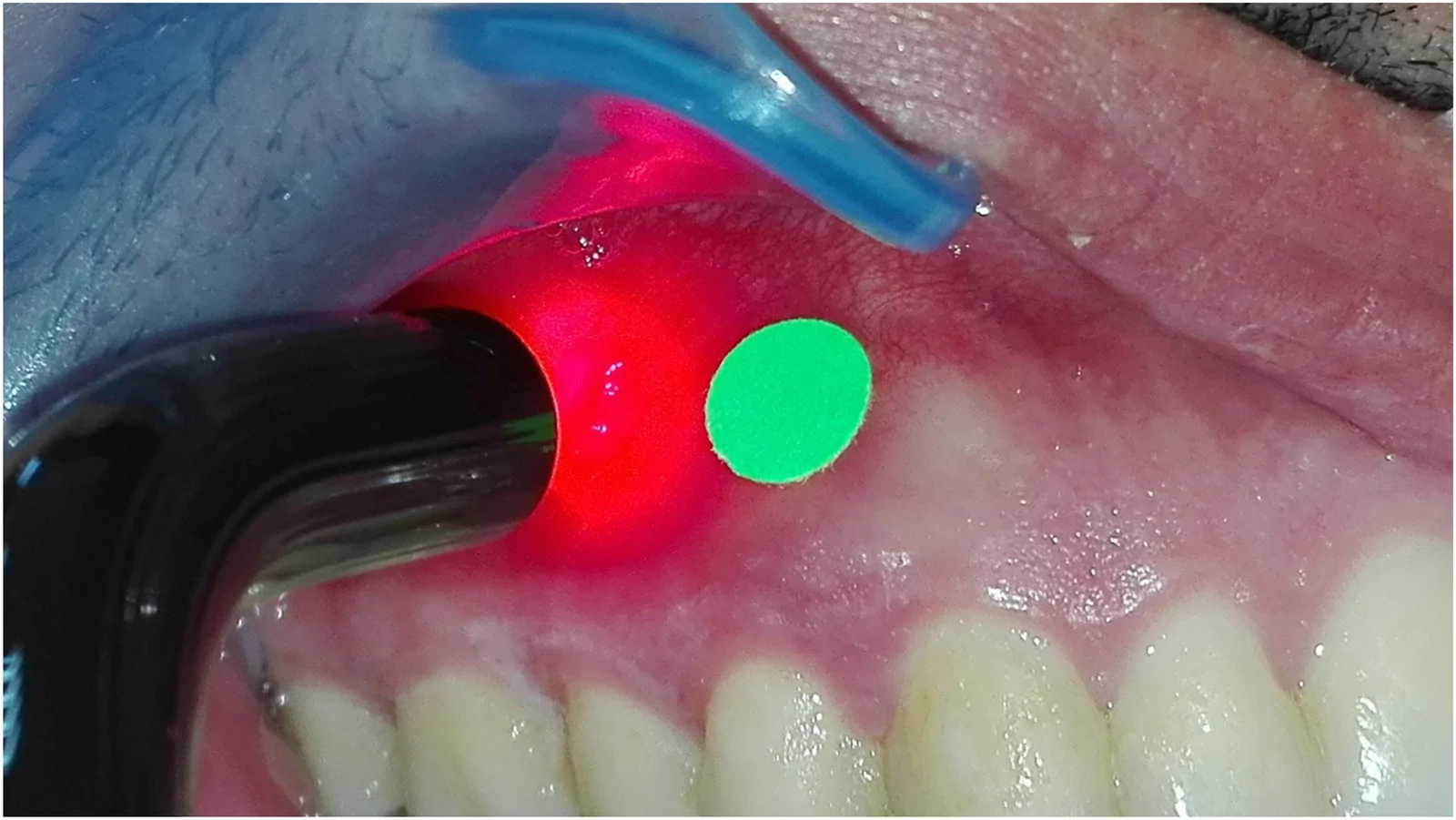
Photobiomodulation therapy application for recurrent aphthous stomatitis.

#### **Group II**: Management with 0.3% apitoxin gel

Participants in group II received topical treatment with 0.3% apitoxin gel, which was applied using a sterile applicator under strict aseptic conditions. Before each application, the ulcer area was gently cleansed with sterile saline to remove any debris or exudate. A thin and even layer of the gel was then applied to the ulcer bed and surrounding mucosa to ensure full coverage.

The gel's adhesive properties promoted prolonged retention on the wound surface, supporting the sustained release of bioactive compounds and maintenance of a moist environment conducive to tissue repair.^28^ The treatment was administered three times daily from day 1 to day 5 to ensure a continuous therapeutic effect. Illustrations of the gel application procedure are provided in Figure 4, Figure 5.^28^^,^^29^

**Figure 4:**
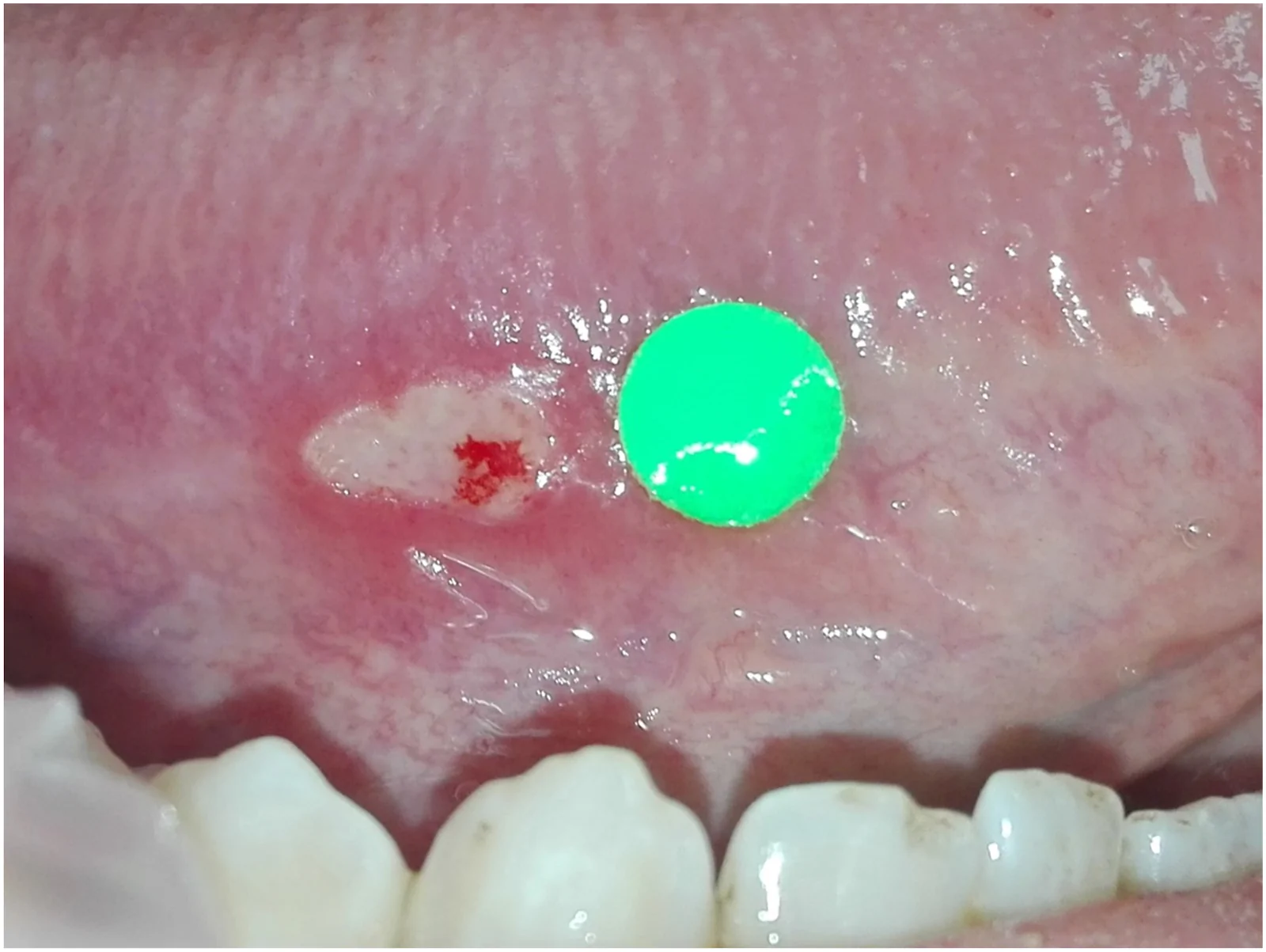
Recurrent aphthous stomatitis before apitoxin application.

**Figure 5:**
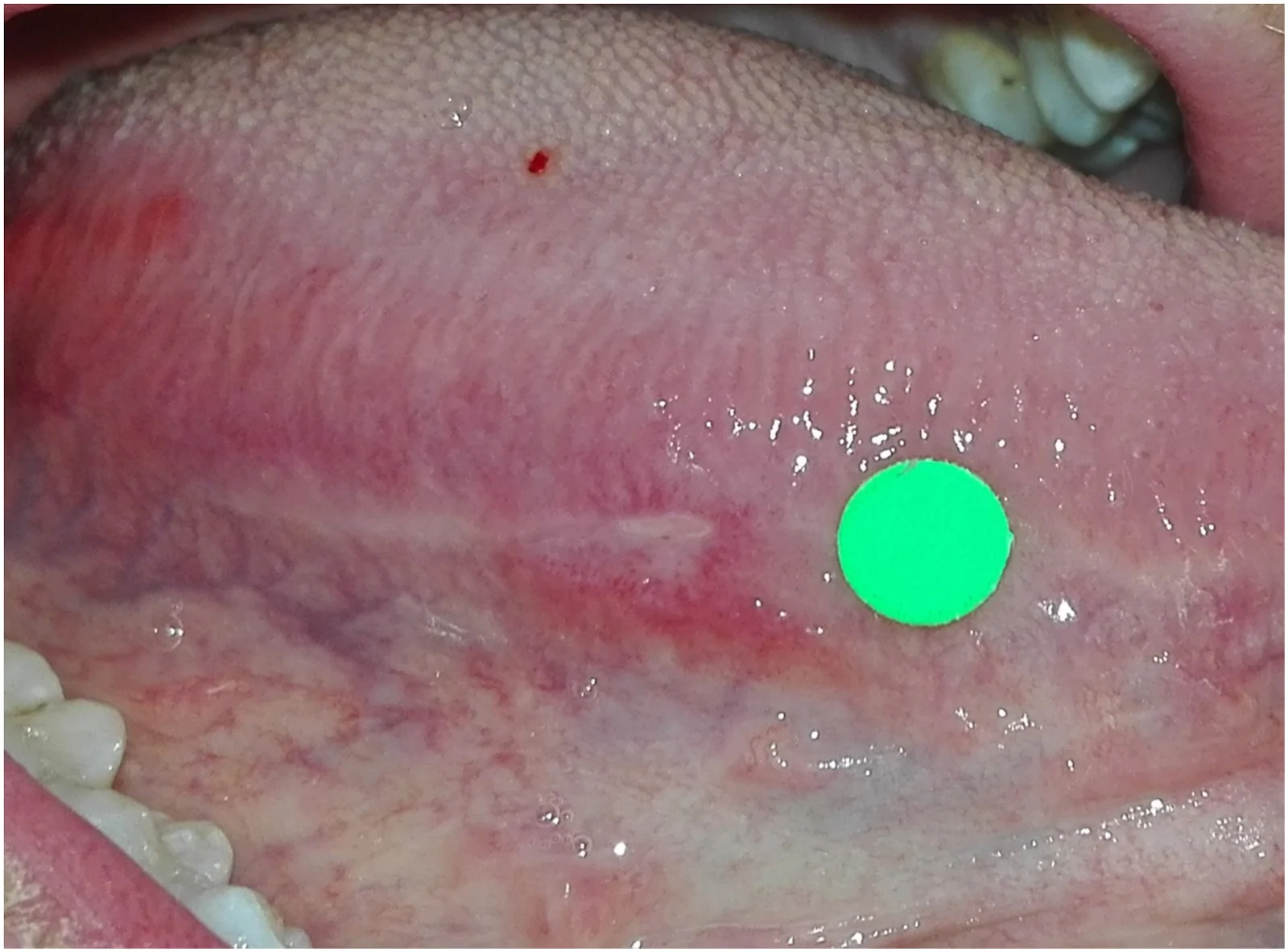
Effect following apitoxin application for recurrent aphthous stomatitis.

### Assessment methods

1. Degree of pain sensation: VAS and EI for pain improvement
2. Change in ulcer size
3. Changes in ulcer depth

#### VAS

A VAS was used to evaluate the degree of pain sensation.

**Figure:**
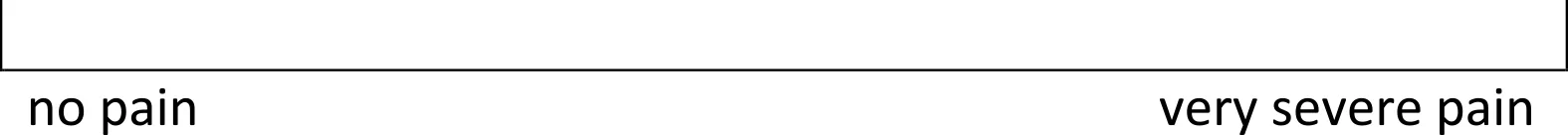

A scale with a length of 100 mm was presented to the patient where 0 represented no pain and 100 represented very severe pain, and the degree of pain was determined from 0 to 100. The number selected by the patient was the VAS score.^28^

The degree of pain sensation was evaluated at:

Day 1: V1, VAS score at baseline;

Day 5: V5, VAS score after complete laser application.

#### EI for pain improvement

•The EI for pain improvement was calculated for each patient with the following formula:^6^

(3) EI = [(V1 –V5)/V1] × 100%.

The EI was evaluated on the following scale with four rankings:^30^

Healed: EI ≥ 95%;

Marked improvement: 70% ≤ EI < 95%;

Moderate improvement: 30% ≤ EI < 70%;

No improvement: EI < 30%.

#### Changes in size of ulcers

Changes in lesion size were recorded digitally. Images were opened using ImageJ software (Version 1.53t; National Institutes of Health), and measurement calibration was performed using the Set Scale option based on the green label (green circle diameter). The ulcer's outline was then demarcated and the ulcer's surface area was measured using the Measure function in ImageJ (National Institutes of Health).

#### Changes in depth of ulcers

Ulcer depth was measured by analyzing pre- and post-treatment images using the gray-level light–shadow profiles generated with ImageJ software. Images were first converted into 32-bit format and a straight line was drawn across each ulcer. The Plot Profile and Surface Plot tools were then used to generate histograms of gray levels, with the highest value corresponding to the ulcer's deepest point. This procedure was repeated for post-treatment images to assess changes in ulcer depth.

### Statistical analysis

IBM SPSS software (Version 24.0; IBM, Armonk, NY, USA) was used to perform statistical analyses of all data. The Shapiro–Wilk test was applied to check the normality of data distributions and the *t*-test for intergroup comparisons. The mean, standard deviation, and *P*-value were calculated. Statistically significant differences were accepted at *P* < 0.05.

## Abbreviations

RAS: Recurrent aphthous stomatitis; PBM: Photobiomodulation; ATP: Adenosine triphosphate; BV: Bee venom VAS: Visual Analog Scale; EI: Effectiveness Index.

## Results

The baseline demographic characteristics of the study participants did not differ significantly between the two treatment groups, confirming their homogeneity. The mean age of the participants was 27.8 ± 6.4 years in group I and 28.5 ± 5.9 years in group II (*P* = 0.68). Group I comprised 16 males (53.3%) and 14 females (46.7%), and group II contained 17 males (56.7%) and 13 females (43.3%). Statistical analysis demonstrated that the gender distribution was well-balanced across both groups with no significant difference (*P* = 0.79) (Table 1).

**Table 1:** Distribution of ulceration prevalence and types across study groups – percentage analysis.

Category	Group I: PBM	Group II: Apitoxin gel
**Prevalence**		
Labial mucosa	58.3%	40%
Buccal mucosa	8.3%	10%
Ventral surface of the tongue	16.7%	10%
Dorsum of the tongue	8.3%	20%
Attached gingiva	8.3%	20%
**Types**		
Major ulcer	25%	40%
Minor ulcer	75%	60%
Herpetiform ulcer	0%	0%

### Pain sensation (VAS)

The VAS scores decreased significantly in both groups from baseline to day 5 (*P* < 0.001). The mean VAS score in group I (PBM) decreased from 9.2 ± 0.89 at baseline to 0.97 ± 1.03 on day 5. The mean VAS score in group II (apitoxin gel), decreased from 9.03 ± 1.47 at baseline to 0.30 ± 0.75 on day 5. The intergroup comparison indicated no statistically significant difference in the VAS scores on day 5 (*P* = 0.211) (Table 2).

**Table 2:** Comparison between the two studied groups according to different parameters.

*Parameter*	*Group*	*Baseline*	*Post-treatment*	*U*	*p*	*Cohen's d*	*95% CI*	*Z*	*p_0_*
***Surface area***	PBM (n = 30)	Min–Max: 2.40–46.90	Min–Max: 0.20–9.70	444.50	0.935	0.32	[−0.45, 1.69]	4.782^a^	**<0.001**^a^
		**Mean ± SD: 14.08**±**9.90**	**Mean ± SD: 3.16**±**2.47**						
		Median (IQR): 12.0 (6.50–19.30)	Median (IQR): 2.50 (1.20–4.80)						
	Apitoxin (n = 30)	Min–Max: 4.34–21.29	Min–Max: 0.49–9.45				4.782	4.782	**<0.001**^a^
		**Mean ± SD: 12.19**±**4.88**	**Mean ± SD: 3.78**±**2.57**						
		Median (IQR): 11.75 (8.16–15.75)	Median (IQR): 2.70 (1.70–6.0)						
***VAS***	PBM (n = 30)	Min–Max: 7.0–10.0	Min–Max: 0.0–3.0	223.50	0.211^a^	0.45	[−0.12, 0.53]	4.836^a^	**<0.001**^a^
		**Mean ± SD: 9.20**±**0.89**	**Mean ± SD: 0.97**±**1.03**						
		Median (IQR): 9.0 (9.0–10.0)	Median (IQR): 1.0 (0.0–2.0)						
	Apitoxin (n = 30)	Min–Max: 6.0–10.0	Min–Max: 0.0–3.0				4.810^a^	4.810^a^	**<0.001**^a^
		**Mean ± SD: 9.03**±**1.47**	**Mean ± SD: 0.30**±**0.75**						
		Median (IQR): 8.0 (7.0–9.0)	Median (IQR): 0.0 (0.0–0.0)						
***Ulcer depth***	PBM (n = 30)	Min–Max: 135.0–265.0	Min–Max: 54.0–247.0	253.50	0.114^a^	0.28	[−2.15, 27.81]	4.484^a^	**<0.001**^a^
		**Mean ± SD: 195.5**±**36.82**	**Mean ± SD: 85.0**±**50.30**						
		Median (IQR): 193.0 (167.0–214.0)	Median (IQR): 113.5 (88.0–165.0)						
	Apitoxin (n = 30)	Min–Max: 72.77–239.0	Min–Max: 25.0–156.0				4.700^a^	4.700^a^	**<0.001**^a^
		**Mean ± SD: 156.4**±**48.48**	**Mean ± SD: 72.17**±**35.23**						
		Median (IQR): 162.0 (114.0–195.0)	Median (IQR): 56.50 (45.0–91.0)						

**U**: Mann–Whitney U test (between-group comparison).

**Z**: Wilcoxon signed-rank test (within-group comparison).

**p**: Between-group p-value.

**p_0_**: Within-group p-value (baseline vs. post-treatment).

**IQR**: Interquartile range; **SD**: Standard deviation.

a Statistically significant (p ≤ 0.05).

### Ulcer size (surface area)

Significant reductions in the ulcer surface area were found in both groups from baseline to day 5 (*P* < 0.001). In group I, the mean ulcer surface area decreased from 12.5 ± 2.1 mm^2^ at baseline to 4.3 ± 1.5 mm^2^ on day 5. In group II, the mean ulcer surface area decreased from 12.8 ± 2.0 mm^2^ at baseline to 3.9 ± 1.2 mm^2^ on day 5. The intergroup comparison detected no statistically significant difference in ulcer surface area reduction between groups I and II on day 5 (*P* = 0.21) (Table 2).

### Ulcer depth

In both groups, the ulcer depth decreased significantly from baseline to day 5 (*P* < 0.001). The mean ulcer depth in group I decreased from 2.1 ± 0.4 mm at baseline to 0.8 ± 0.2 mm on day 5. In group II, the mean ulcer depth decreased from 2.2 ± 0.3 mm at baseline to 0.4 ± 0.1 mm on day 5. The intergroup comparison indicated a statistically significant difference in ulcer depth reduction on day 5, with a greater reduction in group II compared with group I (*P* < 0.001) (Table 2).

### EI

The EI for pain improvement was significantly higher in group II (apitoxin gel) compared with group I (PBM) (*P* < 0.001). The mean EI was 79.5% ± 8.2% in group II and 61.1% ± 7.5% in group I, indicating that apitoxin gel provided superior pain relief (Table 3).

**Table 3:** Comparison of efficacy index (EI%) between groups.

*Parameter*	*Group*	*Min–Max*	*Mean ± SD*	*Median (IQR)*	*U*	*p*
**EI (%)**	PBM (n = 30)	66.60–100.0	**89.86**±**10.87**	90.0 (80.0–100.0)	275.50	**0.003**^a^
	Apitoxin (n = 30)	70.0–100.0	**96.67**±**8.33**	100.0 (100.0–100.0)		

**U**: Mann–Whitney U test (between-group comparison).

**p**: Between-group p-value.

**SD**: Standard deviation.

a Statistically significant (p ≤ 0.05).

### Cytotoxicity test

The cytotoxicity profile obtained for the apitoxin–carbopol gel indicated a concentration dependent but non-lethal effect on human oral epithelial cells. The formulation exhibited high cytocompatibility during the 72-h period, where the cell viability remained above 90% at concentrations of 25% or less, i.e., 90.1 ± 2.4% at 72 h, and above 80% at a concentration of 50%, i.e., 82.9 ± 2.7% at 72 h. There were no significant differences in viability compared with the negative control at all formulation concentrations, including 50%, i.e., *P* > 0.05. Even at the highest formulation concentration, i.e., 100% or undiluted gel, the cell viability remained at 72.7 ± 3.0% after 72 h, with an IC_50_ value of more than 100%. However, the 10% dimethyl sulfoxide positive control exhibited severe toxicity with viability of 10.8 ± 1.2%, thereby validating the sensitivity of the assay and relative safety of the formulation.

## Discussion

In this study, apitoxin gel was investigated as a treatment modality for RAS and compared with PBM. The results indicated that the innovative apitoxin formulation was safe and effective for oral use, highlighting its transformative potential for use in oral healthcare. Our findings indicated that both treatments significantly improved the clinical parameters, including the pain sensation, ulcer surface area, and ulcer depth. In particular, apitoxin gel exhibited superior efficacy at reducing the pain intensity and ulcer depth compared with PBM (Table 4, Table 5).

**Table 4:** Normality Check using Shapiro–Wilk test for the data sets pre and post regarding to different parameters.

Variables	Periods	Group	Shapiro–Wilk		
			Stat.	df	Sig.
Area	Baseline	Apitoxin	0.960	30	0.309
		Soft laser	0.878	30	0.003^a^
	Post treatment	Apitoxin	0.893	30	0.006^a^
		Soft laser	0.914	30	0.019^a^
Pain improvement	First day	Apitoxin	0.871	30	0.002^a^
		Soft laser	0.802	30	<0.001^a^
	Fifth day	Apitoxin	0.466	30	<0.001^a^
		Soft laser	0.818	30	<0.001^a^
Ulcer depth (gray level)	Pre	Apitoxin	0.854	30	<0.001^a^
		Soft laser	0.954	30	0.215
	Post	Apitoxin	0.896	30	0.007^a^
		Soft laser	0.943	30	0.112
EI (%)		Apitoxin	0.460	30	<0.001^a^
		Soft laser	0.835	30	<0.001^a^

a Statistically significant at p ≤ 0.05.

**Table 5:** Multiple Comparison correction using Holm-Bonferroni Sequential Correction.

Variables	Periods	Un-adjustp-value	Adjustp-value
Area	Baseline	0.93518	0.93518
	Post treatment	0.25793	0.51586
Pain improvement	First day	0.00054^a^	0.00325^a^
	Fifth day	0.00233^a^	0.01166^a^
Ulcer depth(gray level)	Pre	0.00366^a^	0.01166^a^
	Post	0.00002^a^	0.00013^a^
EI (%)		0.00278^a^	0.01166^a^

a Statistically significant at p ≤ 0.05.

Thus, the biocompatibility of the apitoxin gel has a considerable degree of clinical significance for aphthous ulceration treatment because the apitoxin gel conforms to the ISO 10993-5^31^, ^32^ criteria for cytotoxicity testing, where a decrease in cell viability below 30% is indicative of non-cytotoxicity. The cell viability rate was above 70%, even at a concentration of 100%, demonstrating the non-cytotoxic nature of the synergistic combination of BV apitoxin and Carbopol Ultrez 30. Similarly, a study conducted by Kurek-Górecka et al.^18^ concluded that the anti-inflammatory effect of BV at a controlled dose did not induce apoptosis in mucosal cells. In addition, the cell viability rate did not change significantly from 24 h to 72 h, indicating the absence of cumulative toxicity, which is a major factor that needs to be considered in the treatment of aphthous ulcers because any treatment would be repetitive (Table 6).

**Table 6:** Effect of serial concentrations of apitoxin gel on the viability of immortalized human oral epithelial cells at 24, 48, and 72 hours, as determined by the MTT assay.

Concentration (%)	Viabilityat 24 h(% ± SD)	Viabilityat 48 h(% ± SD)	Viabilityat 72 h(% ± SD)
Negative control	100 ± 2.1	100 ± 2.0	100 ± 1.9
6.25%	98.4 ± 2.3	97.9 ± 2.1	97.2 ± 2.0
12.5%	95.7 ± 2.5	95.1 ± 2.4	94.4 ± 2.2
25%	91.8 ± 2.8	90.9 ± 2.6	90.1 ± 2.4
50%	84.6 ± 3.0	83.8 ± 2.9	82.9 ± 2.7
100%	74.9 ± 3.3	73.8 ± 3.1	72.7 ± 3.0
Positive control(10% DMSO)	12.1 ± 1.4	11.4 ± 1.3	10.8 ± 1.2

In the present study, apitoxin gel significantly reduced the pain intensity, ulcer depth, and healing time compared with baseline, and in some parameters relative to PBM. These effects can be attributed to the bioactive components of BV. The apitoxin gel probably accelerated mucosal healing and reduced nociceptive signaling, resulting in improved patient-reported pain outcomes.^33^

PBM also produced notable clinical improvements, consistent with previous studies of applying PBM to oral mucosal lesions. PBM operates primarily through mitochondrial stimulation, enhancing ATP production, modulating reactive oxygen species, and activating transcription factors involved in cellular repair and proliferation. These cellular effects translate clinically into faster epithelial regeneration, reduced inflammation, and decreased ulcer-associated pain.^34^

The observed differences in specific outcomes between the two treatment groups may reflect their distinct yet complementary mechanisms of action. Apitoxin gel exerts its effects through biochemical modulation of the inflammatory and immune response, whereas PBM primarily enhances cellular energy metabolism and tissue repair processes. These mechanisms provide a plausible explanation for the differences in clinical efficacy observed in this study.^34^

The observed improvements in pain sensation and ulcer healing in both groups align with previous research.^35^ The significant reduction in pain sensation observed with apitoxin gel (group II) is a crucial finding. This superior analgesic effect can be attributed to the multifaceted mechanisms associated with apitoxin, particularly the actions of melittin and apamin. Melittin inhibits pro-inflammatory cytokines and nuclear factor κB activation, directly reducing inflammation and pain.^35^^,^^36^ Apamin further modulates immune signaling, contributing to pain suppression.^37^ PBM also provides pain relief by modulating inflammatory mediators and nerve activity,^38^, ^39^, ^40^ but the direct pharmacological action of apitoxin may provide a more pronounced analgesic effect in RAS management.

Apitoxin gel also demonstrated superior efficacy at reducing the ulcer depth, possibly due to apitoxin's ability to actively facilitate tissue regeneration by enhancing fibroblast and keratinocyte proliferation, which are essential for restoring damaged oral mucosa.^18^^,^^19^ The sustained release of bioactive components from the gel formulation combined with its adhesive properties probably contributed to maintaining an optimal moist wound environment, further promoting deeper tissue healing. PBM promotes tissue regeneration but the direct cellular proliferative effects of apitoxin appear to have provided an added advantage in reducing the ulcer depth.

Interestingly, there was no significant difference between groups in terms of ulcer surface area reduction. This suggests that both PBM and apitoxin gel were equally effective at promoting superficial wound closure. However, the superior pain and ulcer depth outcomes with apitoxin gel highlight its comprehensive therapeutic benefits beyond mere surface healing.

Our results are consistent with those obtained by Aggarwal et al. (2014) who reported that PBM provided immediate and complete pain relief for recurrent aphthous ulcers, highlighting its efficacy in reducing the ulceration size and healing time.^41^ Similarly, Lalabonova and Daskalov^42^ observed significant reductions in pain and inflammation with PBM, as well as accelerated epithelialization in aphthous ulcers. These studies reinforce the role of PBM as an effective and non-invasive treatment option for RAS management. In addition, laser therapy alters the electrical activity in nerve cells, further contributing to its analgesic properties.^38^^,^^39^^,^^43^ However, few previous studies have investigated laser therapy for aphthous ulcers, and thus comparative insights between laser and pharmacological treatments are lacking.^38^

This study is the first to compare the effectiveness of apitoxin and PBM therapy for RAS treatment. The results highlight the possible use of apitoxin as a natural, cost-effective, and accessible alternative, with great potential as a novel therapeutic agent. These findings align with the global trend of integrating natural products into medical practices, and apitoxin is a promising candidate for further investigation. Future research is essential to obtain more substantial evidence to support the use of apitoxin and laser therapy in managing oral ulcerations and related conditions.

The present study also had some limitations, such as variability in individual wound-healing rates and the inability to achieve operator blinding due to the different application methods, which may have introduced bias. Moreover, the lack of long-term follow-up prevented evaluation of the sustained therapeutic effects of apitoxin, and thus further studies with larger, controlled, and blinded designs are recommended.

## Conclusion

The results obtained in the present study showed that apitoxin gel effectively reduced pain, the ulcer surface area, and ulcer depth, and accelerated healing in RAS. Clinically, apitoxin gel is a promising alternative or adjunctive treatment for RAS, providing faster symptom relief and improved mucosal recovery. Further *in vitro* and *in vivo* studies are needed to elucidate the precise molecular mechanisms involved and optimize its clinical application.

## Author contributions

Conceptualization, RSS, SMMG, and MOE; methodology, RSS, SMMG, and MOE; software, MOE and MIF; validation, MIF, AA, and RM; formal analysis, MIF, AA, and RM; investigation, MIF, AA, and MOE; data curation, MIF; writing—original draft preparation, RSS, SMMG, and MOE; writing—review and editing, MIF, AA, and RM; visualization, AA and RM; supervision, MOE

## Ethics approval and consent to participate

The research ethics committee at the College of dentistry, Taibah University, ethically approved this study, with reference No, TUCDREC/300424/Rsabra.

All participants approved to participate in the study and signed a consent form.

This human study were conducted in accordance with the applicable laws, ethical guidelines, and regulations governing human research. Approval was obtained from the relevant ethical review board, and informed consent was obtained from all participants prior to their inclusion in the study.

## Consent for publication

All participants signed informed consent forms to publish their data and images.

## Availability of data and materials

Data available upon responsible request from the corresponding author.

## Funding

This research did not receive any specific grant from funding agencies in the public, commercial, or not-for-profit sectors.

## Conflict of interest

The authors have no conflicts of interest to declare.
